# Pandemic-Driven Disruptions in Oral Health: Transformative Trends in Care for Older Adults

**DOI:** 10.1093/geroni/igab046.1601

**Published:** 2021-12-17

**Authors:** Samuel Zwetchkenbaum, Mary Fisher, Brooke Fukuoka, Jeffrey Dodge, Michael Helgeson, Elisa Ghezzi

**Affiliations:** 1 Rhode Island Department of Health, Providence, Rhode Island, United States; 2 Michigan Geriatric Dental Care, West Bloomfield, Michigan, United States; 3 Your Special Smiles PLLC, Jerome, Idaho, United States; 4 CareLink, Woonsocket, Rhode Island, United States; 5 Apple Tree Dental, Mounds View, Minnesota, United States; 6 University of Michigan School of Dentistry, Ann Arbor, Michigan, United States

## Abstract

Oral healthcare for the aging was severely disrupted during the pandemic of coronavirus disease 2019 (COVID-19). Transformative changes in care delivery involved teledentistry, mobile/portable dentistry, minimally invasive dentistry, aerosol minimization, and interprofessional oral care. Management of chronic oral health problems evolved through periods of limited to no access to daily and professional oral healthcare. Access to care has been influenced by availability of the oral care workforce, variability in long term care policy, and the lack of funding to cover medically necessary services delivered via asynchronous telehealth technologies. Impacts were identified six and twelve months into the pandemic. These will be compared to the state of oral healthcare for the aging 18 months from the start of the pandemic. The impact of vaccination on access to care will be explored. Variability between states (Idaho/Michigan/Minnesota/Rhode Island) will be addressed. Directions of new and needed research opportunities will be discussed.

